# Antimicrobial and mechanism of antagonistic activity of *Bacillus* sp. A2 against pathogenic fungus and bacteria: The implication on honey's regulatory mechanism on host's microbiota

**DOI:** 10.1002/fsn3.1770

**Published:** 2020-07-20

**Authors:** Lina Jia, Janet Cheruiyot Kosgey, Jielin Wang, Jianxun Yang, Rose Magoma Nyamao, Yi Zhao, Xue Teng, Lei Gao, MartinTherese Cheteu Wabo, Natalia V. Vasilyeva, Yong Fang, Fengmin Zhang

**Affiliations:** ^1^ Department of Microbiology WU Lien‐Teh Institute Harbin Medical University Harbin China; ^2^ Department of Dermatology The 2nd Hospital of Harbin Medical University Harbin China; ^3^ School of biological and life sciences The Technical University of Kenya Nairobi Kenya; ^4^ School of Medicine Kenyatta University Nairobi Kenya; ^5^ Department of Microbiology Kashkin Research Institute of Medical Mycology North‐Western State Medical University named after I.I. Machnikov Saint Petersburg Russia

**Keywords:** antagonistic bacteria, antimicrobial activity, *Bacillus* sp, honey, pathogenic fungus

## Abstract

Honey is thought to act against microbes and regulates microbiota balance, and this is mainly attributed to the enzymatic production of hydrogen peroxide, high osmolarity, and nonperoxidase factors, for example, lysozyme and botanical sources of nectar, while the effect of honey's probiotic is recently considered. The study of honey as source of beneficial microbes is understudied. The purpose of this study was to screen for the beneficial microorganisms in honey with antagonistic property against important pathogens and the mechanism of antimicrobial activity and thus play a beneficial role as probiotics. The results showed that one out of the fourteen bacterial isolates had antimicrobial activity and was identified as *Bacillus* Sp. A2 by 16S rRNA sequence and morphology. Antimicrobial activity of the isolate against *C. albicans*, *E. coli*, and *S. aureus* was confirmed by Agar well diffusion and liquid coculture assays, and the propagation of those microbes was significantly inhibited after treatment with the isolate *Bacillus* sp. A2 (*p* < .05) in comparison with untreated negative control and positive control (fluconazole, chloramphenicol, *L. plantarum*). The morphological changes including the distorted shape with indentations and leakages (*SEM*), damaged cell membrane, and cell wall with the disintegration and attachment of the *Bacillus* sp. A2 (TEM) in treated *C. albicans* were observed. Meanwhile, reactive oxygen species accumulation and decreased mitochondrial membrane potential were detected in treated *C. albicans*. These results revealed that the isolate *Bacillus* sp. A2 from honey has significant antimicrobial activity (*p* < .05) against *C. albicans* in comparison with untreated negative control and positive control *L. plantarum*, which depends on the accumulation of reactive oxygen species, mitochondrial damage, and the cell apoptosis. We concluded that the *Bacillus* sp. A2 possess the antimicrobial property, which may contribute to regulation of host's microbiota as a beneficial microbe or probiotic.

## Graphical abstract

Antimicrobial activity of *B. amyloliquefaciens* A2 from honey against *C. albicans*, *C. glabrata*, *E. coli*, and *S. aureus* in vitro. *B. amyloliquefaciens* A2 from honey induce ROS accumulation, mitochondrial damage, and cell apoptosis in *C. albicans*. It is implicated that the beneficial microbe or probiotics in honey is contributed to the honey's antimicrobial activity and regulation of host's microbiota balance.

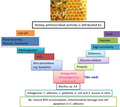

## 1 INTRODUCTION

Microbiota is composed of abundant microbes including bacteria, fungi, viruses, and helminths. The homeostasis of microbiota is important for the host health and related disease, which is regulated by drug, prebiotic, probiotic, and others.

Honey is documented in the most ancient literature for its medicinal value (Mandal & Mandal, 2011) and is recently considered to act against infection and regulate host microbiota (Hussain & Medicine, 2018; Miguel, Antunes, & Faleiro, 2017; Olofsson et al., 2016). This is because honey is rich in sugars, several vitamins (B complex, vitamin C, ascorbic acid, pantothenic acid, niacin, and riboflavin), and minerals (calcium, copper, iron, magnesium, manganese, phosphorus, potassium, and zinc) (Vallianou, 2014). Different samples of honey have varying degrees of antimicrobial activity, which is associated with the botanical sources of nectar (Koc et al., 2009; Mandal & Mandal, 2011; Matzen et al., 2018; Moussa, Noureddine, Saad, Abdelmelek, & Abdelkader, 2012). Some of the factors attributed to antimicrobial property of honey are documented (Mandal & Mandal, 2011), that is, the enzymatic production of hydrogen peroxide (H_2_O_2_), low pH and high osmolarity, and nonperoxidase factors, for example, lysozyme. However, Wahdan et al. (1998) observed that fungal pathogens *Candida* and *Trichophyton* were much more tolerant to the high concentration of sugar in honey compared to bacteria; therefore, sugar concentration is not attributed to antifungal property of honey. Therefore, other than its rich composition of nutrients and high sugar, there are other antimicrobial factors.

Several beneficial microorganisms with antimicrobial activity have been isolated from honey (Amin et al., 2020; Hussain & Medicine, 2018; Keerthi 2018 ). Among them are *Paenibacillus polymyxa* TH13 with anti‐*Paenibacillus* larvae species activity, *B. amyloliquefaciens* with anti‐*B. dothidea* activity (Li et al., 2016), and lactic acid bacteria with anti‐*Candida* spp. activity (Bulgasem, Lani, Hassan, Wan Yusoff, & Fnaish, 2016). Other than nutrition, food can provide us with beneficial microorganisms that protect our bodies from disease by creating a barrier and competing with pathogens for nutrition and binding sites. These microorganisms can also produce antimicrobial compounds that inhibit pathogens (Amara & Shibl, 2015; V. H. Matsubara, Bandara, Mayer, & Samaranayake, 2016). Therefore, honey is a good candidate food that can be source of probiotics. However, there is lack of knowledge on the mechanism of antimicrobial activity of these beneficial microbes.

This study investigated antimicrobial activity of the beneficial microorganisms in honey against important fungi and bacteria pathogens, as well as the mechanism of the antimicrobial activity, with the goal of being source of probiotic.

## 2 MATERIAL AND METHODS

### 2.1 Strain, media, culture conditions, and chemicals

*C. albicans* ATCC10231, *E. coli* CGMCC25922, *S. aureus* CGMCC25923, and *L. plantarum* CGMCC1.12974 were obtained from China General Microbiological Culture Collection (CGMCC), and the honey isolate was from Western Australia Jarrah Honey (AOMI PTY Co. Ltd., Australia). *C. albicans* ATCC10231 (10^5^ cfu/ml) were cultured in yeast peptone dextrose (YPD) broth and Sabouraud dextrose agar (SDA) slants and *E. coli* 25,922 (10^8^ cfu/ml), *S. aureus* 25,923 (10^8^ cfu/ml), and bacterial honey isolate (10^8^ cfu/ml) in nutrient broth (NB) or nutrient agar (NA) and bacterial honey isolates (10^8^ cfu/ml). The concentration was counted using hemocytometer, optical density (OD), and confirmed by viable counts (cfu/ml) on agar plate.

Cocultures of bacterial isolate and *C. albicans* ATCC10231 were cultured in a mixture of 1:1 nutrient: YPD broth. Incubation conditions were optimized at 37°C for 24 hr in an aerobic incubator (180rpm for liquid cultures). Bacterial isolate (10^8^) was counted using optical density (OD) and confirmed by viable counts (cfu/ml) on nutrient agar, whereas *C. albicans* (10^5^) were counted using hemocytometer, optical density (OD), and confirmed by viable counts (cfu/ml) on SDA. Fluconazole (5,120 µg/L) (Biotopped Life Sciences) was dissolved in double‐distilled water, while chloramphenicol (1 mg/ml) (Biotopped Life Sciences) was dissolved in methanol. Fluconazole (5,120 µg/L) (Biotopped Life Sciences) was dissolved in double‐distilled water, while chloramphenicol (1 mg/ml) (Biotopped Life Sciences) was dissolved in methanol. Media were purchased from Hopebio (China).

### 2.2 Isolation and identification of microorganisms from honey

This was done according to the method (Lee, Churey, & Worobo, 2008a, 2008b). One gram of honey was inoculated into 99 ml of nutrient broth and serially diluted to obtain single bacterial colonies on plate. The candidate bacterial isolates were selected by antimicrobial activity experiment. Colony morphology was documented on nutrient agar, YPD agar, and nutrient agar supplemented with glucose (20 g/L), yeast extract (4 g/L), manganese sulfate (0.04 g/L), magnesium sulfate (0.2 g/L), and Tween‐80 (1 g/L). Bacterial morphology was assessed using light and electron microscope. One isolate with significant antimicrobial activity was identified using the colony morphology and DNA sequence of PCR‐amplified 16S rRNA fragment. Briefly, the genomic DNA of the honey bacterial isolate was prepared and amplified by PCR using the universal primer pair 516‐*F* (5'‐CCCTCATTTGTGCTCGTGTC‐3') and 1510‐R (5'‐ CCTTCYGCAGGTTCACCTAC‐3') (Li et al., 2016). The sequence of the PCR products was completed (Sangon Biotech), and the 16S RNA sequences were blasted at NCBI to check similarity of other bacterial strains.

### 2.3 Antimicrobial activity screened using agar well diffusion

Agar well diffusion (Kosgey et al., 2019; Shehata, El Sohaimy, El‐Sahn, & Youssef, 2016) was carried out on the agar plates, inoculated evenly with 10 µl of *E. coli* (10^8^ cfu/ml), *S. aureus* (10^8^ cfu/ml), or *C. albicans* (10^5^ cfu/ml) each, and then, 10 µl of honey (50% *v*/*v* in PBS), honey isolate (10^8^ cfu/ml) and PBS was added immediately in the wells of each agar plate. The growth inhibitions of three independent experiments were measured as diameter (mm) of a clear zone around the well after incubation at 37°C for 24 hr, and the results were compared to untreated and drug as controls.

### 2.4 Antimicrobial activity confirmation by liquid coculture

Liquid coculture (Kosgey et al., 2019; Kosgey et al., 2020; Victor Haruo Matsubara, Wang, Bandara, Mayer, & Samaranayake, 2016) was done in order to confirm the antimicrobial activity of honey isolate. The honey isolate (10^8^) was cocultured with *C. albicans* (10^5^ cfu/ml) in NA: YPD (1:1) broth, and *E. coli* (10^8^ cfu/ml) and *S*. *aureus* (10^8^ cfu/ml) in nutrient broth at 37°C for 24 hr in an aerobic incubator (180rpm). Positive control *L. plantarum* was cocultured with *C. albicans* (10^5^ cfu/ml) in YPD (1:1) broth supplemented with glucose (20 g/L), yeast extract (4 g/L), manganese sulfate (0.04 g/L), magnesium sulfate (0.2 g/L), and Tween‐80 (1 g/L), and *E. coli* (10^8^ cfu/ml) and *S*. *aureus* (10^8^ cfu/ml) in supplemented nutrient broth at 37°C for 24 hr in an aerobic incubator (180rpm). One milliliter of the cultures was retrieved, and viable counts of *C. albicans* were selectively cultured on YPD agar with chloramphenicol, while bacteria were cultured on supplemented NA agar at 37°C for 24 hr to 48 hr and colonies were distinguished by color. For *L. plantarum* cocultured, viable colonies were selectively grown in NA, in which the probiotic could not grow. Furthermore, the honey isolate was sampled at 1, 2, 4, 8, 12, 24, 48, and 72 hr, and the growth curve was obtained by measuring the change of OD with time.

Additionally, we investigated the antimicrobial activity of the honey isolate against other pathogens with varying degrees of drug resistance. These were *C. glabrata* (10^5^ cfu/ml), which were obtained from China General Microbiological Culture Collection (CGMCC). Bacterial laboratory strains that were resistant to antibiotic disks of cefotaxime, ceftazidime, cefoperozone, and cefatriaxone (Biotopped Life Sciences) and *E. coli* SYY89 (10^8^ cfu/ml), *E. coli* DR115 (10^8^ cfu/ml), and *P. aeruginosa* (clinical isolate) (10^8^ cfu/ml) were also screened following the method outlined above.

### 2.5 Morphology observation under electron microscopy

Morphology of *C. albicans* with or without the treatment was observed under scanning electron microscopy (*SEM*) and transmission electron microscopy (TEM) (Hitachi, Japan), according to the routine methodology. Cocultures were grown in 1:1 NB:YPD broth (180rpm) at 37°C for 24 hr. Controls were pure cultures of the strains used; sterile coverslips were put inside the cultures. The slides were washed gently two times in 0.1 mol/L PBS (PH 7.0). The slides were then fixed with 2.5% glutaraldehyde in PBS for 2 hr and washed three times for 15 min in 0.1 mol/L PBS at 4°C. Then, the cells were postfixed with 1% osmium tetroxide in PBS for 1 hr and washed three times with 0.1 mol/L PBS at 4°C. Following this, the samples were dehydrated in alcohol (30%, 50%, 70%, 80%, 90%, 100%) each time for 15 min at 4°C, freeze‐drying, and sputtered with gold. Then, we used *SEM* to observe the sample (S3400‐N Hitachi).

The TEM samples were fixed as the *SEM*. Then, the samples were dehydrated after alcohol and infiltrated with acetone and epoxy resin mixture, and ultrathin sections were obtained and were transferred onto copper grids covered with the Formvar membrane. 1% uranyl acetate and lead citrate were used for contrast staining. The sections were photographed with a transmission electron microscope (HT7700, Hitachi).

### 2.6 Reactive oxygen species (ROS) detection in *C. albicans*

Intracellular ROS accumulation in treated *C. albicans* was assessed using dihydrorhodamine‐123 (DHR‐123) (Roche) stain. The positive control was *C. albicans* (10^5^) with 20 mmol/L H_2_O_2_ treatment for 4 hr. The cells were harvested and stained with 10mM DHR‐123 for 30 min at 37°C, and then washed three times with PBS. Immediately after staining, fluorescent intensity was assessed using an Olympus FluoViewFV500/IX laser scanning confocal microscope and quantitative analysis of intracellular ROS (green fluorescence) from three independent experiments using ImageJ software.

### 2.7 Mitochondrial membrane potential

JC‐1 probe (Sigma) was used to ascertain changes in mitochondrial membrane potential (∆Ψm). *C. albicans* (10^5^ cfu/mL) cocultured with honey isolate were stained with 1 mg/ml of JC‐1 at 37°C for 20 min (Kosgey et al., 2020; Ma et al., 2016; Pina‐Vaz et al., 2001). Sodium azide (1mM) (Tianjin Fuchen), a fungal respiratory inhibitor, was used as positive control (Pina‐Vaz et al., 2001). Immediately after staining, the mean of the fluorescence intensities was captured using an laser scanning confocal microscope (Olympus FluoViewFV500/IX) and quantitative analysis of mitochondrial membrane potential (ratio of green/red fluorescence) from three independent experiments using ImageJ software, and the ratio of aggregated JC‐1 (FL2) to monomer of JC‐1 (FL1) intensity was calculated.

### 2.8 Statistical analysis

The data are presented as mean ± standard deviation from three independent experiments using GraphPad Prism 5.01 (GraphPad Software). The statistically significant differences between untreated control and isolate‐treated samples of dose, ROS, and mitochondrial membrane potential assays were subjected to two‐way ANOVA tests, followed by Tukey's multiple comparison tests. The growth curve was drawn using nonlinear regression, Gaussian distribution. A *p*‐value < .05 was considered to be significant, * denoted *p* < .05, ** denoted *p* < .01, *** denoted *p* < .001, and **** denoted *p* < .0001.

## 3 RESULTS

### 3.1 Genetic identification and biological features of the honey bacterial isolate

**Figure 1:**
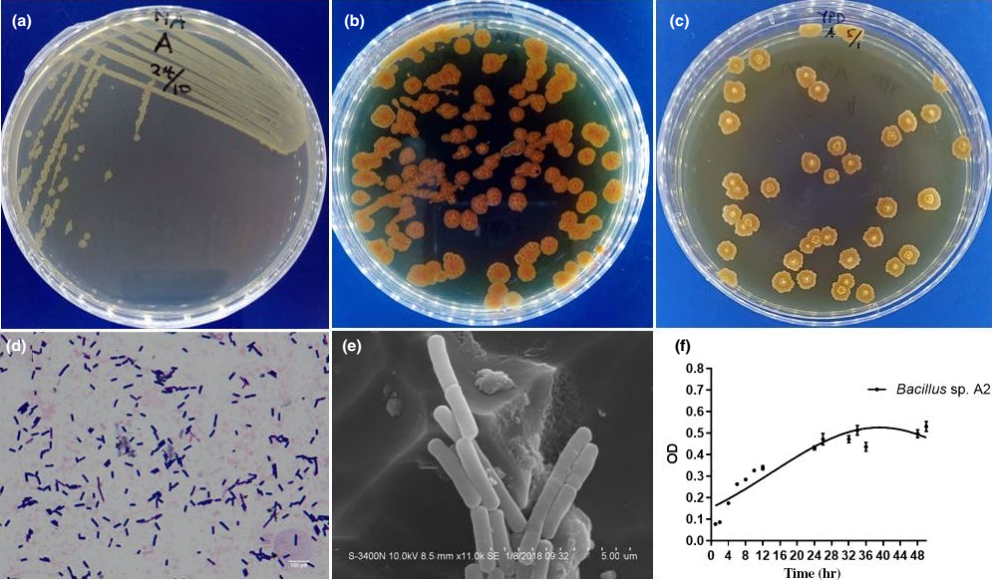
Phenotypic characterization of *Bacillus* sp. A2. Colony morphology on (a) nutrient agar, (b) supplemented nutrient agar (c) YPD, (d) Gram stain (×100 magnification) (scale bar 100µm) phase‐contrast microscope, (e) scanning electron microscopy of A2 (×11,000 magnification) (scale bar 5µm), and (f) illustrates the growth curve of *Bacillus* sp. A2 using optical density (mean ± *SD*) of three independent experiments with three replicates each

The bacterial isolate was analyzed using PCR amplification and sequence of the 16S rRNA. The sequence was blasted in NCBI, and the hits on top with 99% similarity were *Bacillus* strains, predominantly *B. subtilis*/*B. amyloliquefaciens* group. Therefore, the bacterial isolate was identified as *Bacillus* sp. A2. The isolate colony features are flat, nonmucoid, nonpigmented on nutrient agar, but had orange color on supplemented NA and YPD (Figure 1a,b,c). Besides, the bacterial isolate was observed as bacilli under microscopy and showed Gram‐positive (Figure 1d,e). The growth curve of the isolate was made during the culture in YPD (Figure 1f).

### 3.2 Antimicrobial activity detection of the honey and honey bacterial isolates

**Figure 2:**
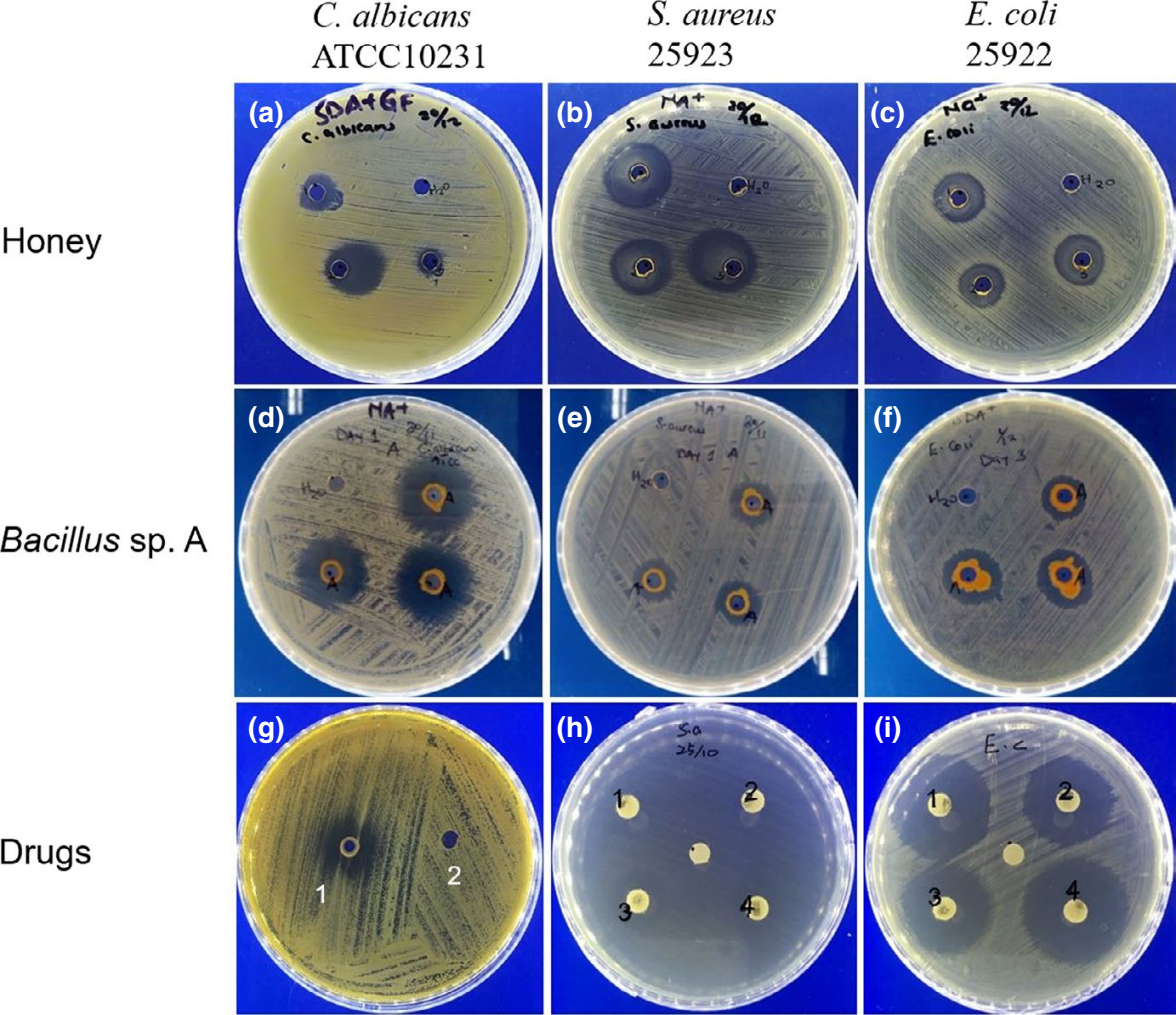
Growth inhibition of honey, *Bacillus* sp. A2, and control drugs using agar well diffusion assay. Honey antimicrobial activity against (a) *C. albicans* ATCC10231 (10.67 ± 1.22), (b) *S. aureus* 25,923 (22.9 ± 0.78), and (c) *E. coli* 25,922 (21.5 ± 1.28). Antimicrobial activity of *Bacillus* sp. A2 against (d) *C. albicans* ATCC10231 (20.50 ± 1.38), (e) *E. coli* 25,922 (11.77 ± 2.31), and (f) *S. aureu*s 25,923 (9.67 ± 1.32 mm). Antimicrobial activity of drugs (g) *C. albicans* ATCC10231 (fluconazole 19.50 ± 1.27), (h) *E. coli* 25,922 (chloramphenicol 21.90 ± 0.50), and (i) *S. aureu*s 25,923 (chloramphenicol 29.10 ± 1.01 mm). The negative control was PBS (labeled H_2_O). The inhibition zone diameters were measured in mm and presented as mean ± *SD* of three independent experiments with three replicates each

The agar diffusion results measured in millimeters (mm) showed that raw honey exhibited statistically significant antimicrobial activity against *S. aureus* 25,923 (22.9 ± 0.78) (*p* < .001), while *E. coli* 25,922 (21.5 ± 1.28) had no statistically significant antimicrobial activity (*p* > .05) in comparison with treated positive control chloramphenicol, which had 2.6 ± 1.50 and 29.00 ± 1.30, respectively. Honey had only a slight antimicrobial activity against *C. albicans* (10.67 ± 1.22), which was statistically different from fluconazole (19.2 ± 1.30) (*p* < .05) (Figure 2). Furthermore, fourteen bacterial isolates were screened for antimicrobial activity. One of them exhibited statistically significant antimicrobial activity against *C. albicans* ATCC10231 (20.50 ± 1.38), which was similar with fluconazole (*p* > .05). The bacterial isolate antimicrobial activity against both *S. aureus* 25,923 (9.67 ± 1.32) and *E. coli* 25,922 (11.77 ± 2.31) was statistically different in comparison with positive control chloramphenicol, which had 2.6 ± 1.12 and 29.00 ± 1.30, respectively (Figure 2).

**Figure 3:**
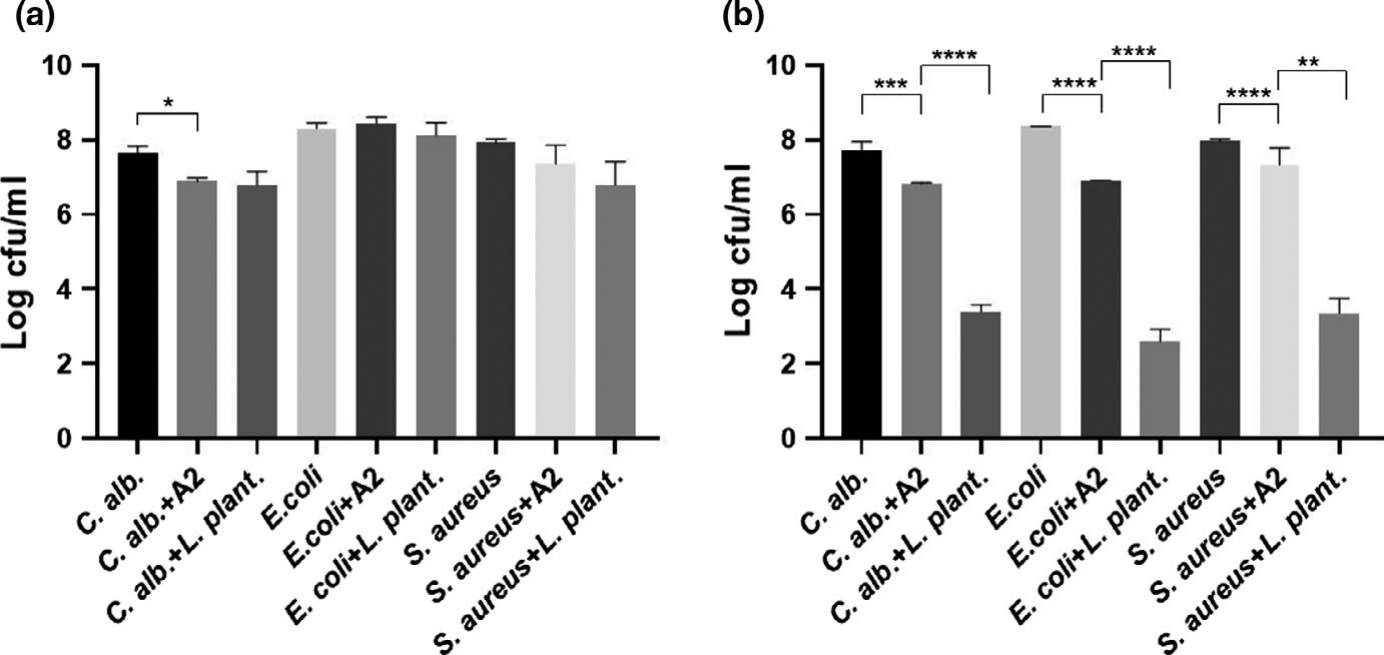
Antimicrobial activity of *Bacillus* sp. A2 confirmed by coculture assay; (a) antimicrobial activity of *Bacillus* sp. A2 (10^8^) in cfu/ml after 24‐hr coculture. (b) 48‐hr coculture against *C. albicans* ATCC10231 (*C. alb*.), *E. coli* 25,922, and *S. aureus* 25,923, respectively, in comparison with control (NC untreated group) and positive control (*L. plantarum* CGMCC 1.12974 10^8^ cfu/ml) of the same microorganism. The values are presented as mean ± *SD* of viable microorganisms in colony‐forming units per ml (cfu/ml) from three independent experiments with three replicates each. A *p*‐value < .05 was considered to be significant, * denoted *p* < .05, ** denoted *p* < .01, and *** denoted *p* < .001

The antimicrobial activity of the isolate was confirmed also by liquid cocultures, which showed significant decreased growth of drug‐susceptible *C. albicans* ATCC10231 after 24 hr of incubation. *C. albicans* ATCC10231, *E. coli* 25,922, and *S. aureus* 25,923 pathogens were all susceptible to the bacterial isolate after 48 hr of incubation in comparison with their untreated control (*p* < .0001) (Figure 3), and it had significantly lower inhibition compared to treated positive control probiotic *L. plantarum* (*p* < .05).

**Figure 4:**
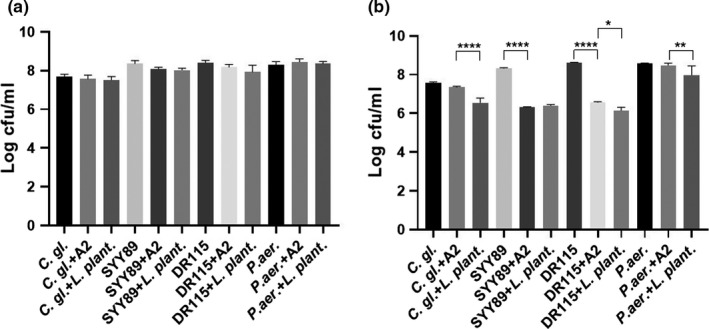
Antimicrobial activity of *Bacillus* sp. A2 against other pathogens by coculture assay; (a) antimicrobial activity of *Bacillus* sp. A2 (10^8^) in cfu/ml after 24‐hr coculture. (b) 48‐hr coculture against *C. glabrata* 2.3983 (C. gl.), *E. coli* SYY89, *E. coli* DR115, and *P. aeruginosa* clinical (*P. aer*.) isolate, respectively, in comparison with control (NC untreated group) and positive control (*L. plantarum* CGMCC 1.12974 10^8^ cfu/ml) of the same microorganism. The values are presented as mean ± *SD* of viable microorganisms in colony‐forming units per ml (cfu/ml) from three independent experiments with three replicates each. A *p*‐value < .05 was considered to be significant, * denoted *p* < .05, ** denoted *p* < .01, and *** denoted *p* < .001

The susceptibility of drug‐resistant pathogens to the bacterial isolate was varied. All the screened pathogens were not susceptible to the bacterial isolate after 24 hr of incubation. However, after 48 hr of incubation *E. coli* SYY89 and DR115 were susceptible to the bacterial isolate in comparison with untreated negative control (*p* < .05). However, the bacterial isolate had significantly lower inhibition compared to positive control probiotic *L. plantarum* (*p* < .05). The exception was against *E. coli* SYY89 in which the activity of the honey bacterial isolate and *L. plantarum* was similar (*p* > .05) (Figure 4).

### 3.3 Morphological observation of *C. albicans* treated under *SEM* and TEM

**Figure 5:**
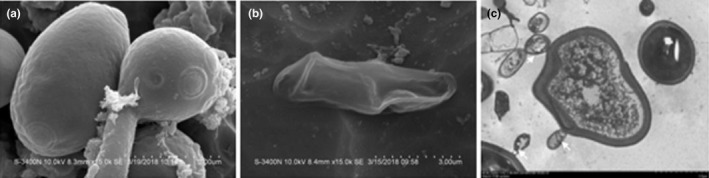
Illustration of morphological changes in electron micrographs of *C. albicans* cocultured with or without *Bacillus* sp. A2. (a) Untreated *C. albicans* (scale bar 3 µm, *SEM*), (b) *C. albicans* cultured with *Bacillus* sp. A2 (scale bar 3 µm, *SEM*), (c) *C. albicans* cultured with *Bacillus* sp. A2, and white arrows point to attached bacteria (scale bar 2 µm, TEM)

Compared to the untreated *C. albicans*, it had even, round/oval shape, turgid cell shape, and budding, and homogenous cell wall (Figure 5a), and the shape of *C. albicans* cocultured with *Bacillus* sp. A2 was distorted and collapsed with indentations and leakages (Figure 5b) as observed under *SEM*. Furthermore, the treated *C. albicans*’ cell membrane and cell wall in TEM micrographs were irregular and damaged with the sign of disintegration and attachment of the *Bacillus* sp. A2, while the organelles were hardly visible (Figure 5c).

### 3.4 Accumulation of reactive oxygen species (ROS) in treated *C. albicans*

**Figure 6:**
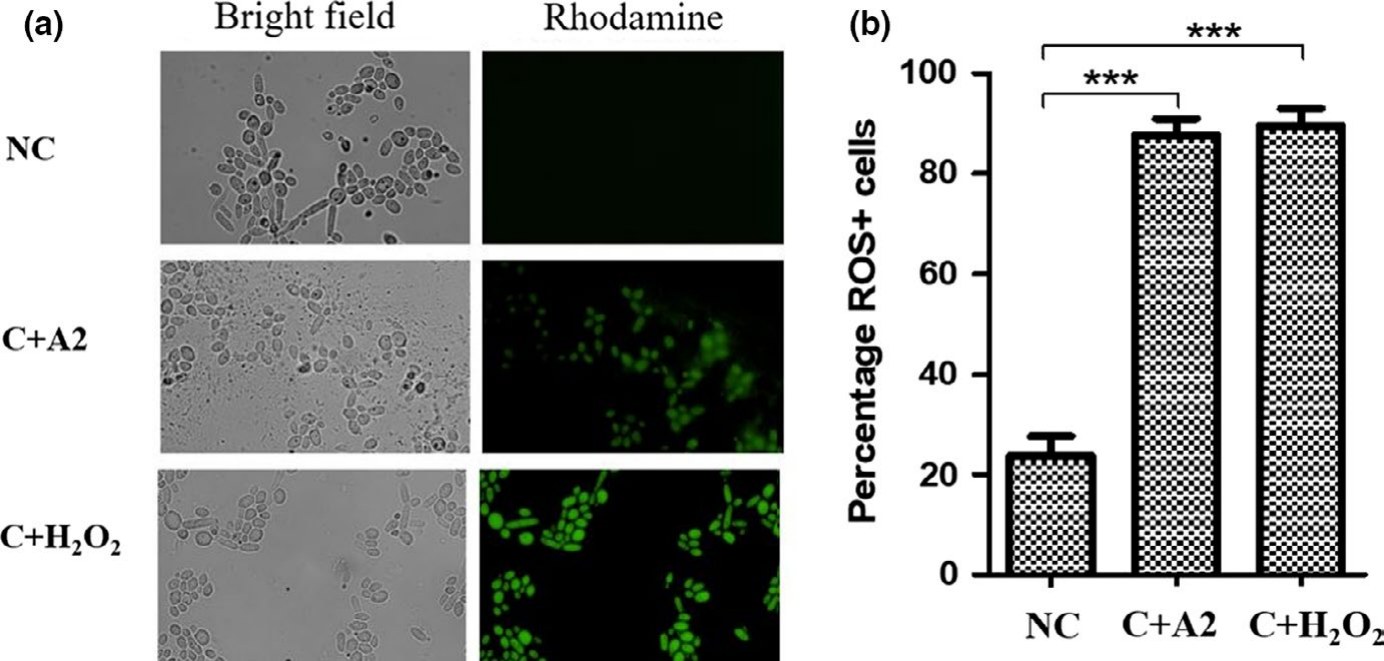
ROS accumulation in treated *C. albicans*. (a) ROS‐positive cells in *C. albicans* with various treatments, and (b) percentage of ROS‐positive *C. albicans* with various treatments stained by rhodamine (DHR‐123) (mean ± *SD*). The experiments were done three independent times, with three replicates each. A *p*‐value < .05 was considered to be significant, * denoted *p* < .05, ** denoted *p* < .01, and *** denoted *p* < .001. Key: NC: untreated *C. albicans* (negative control), C + A2: *C. albicans* treated with *Bacillus* sp. A2; C + H_2_O_2_: *C. albicans* treated with H_2_O_2_ (positive control)

Accumulation of reactive oxygen species (ROS) was detected in the *C. albicans* cell, to explain the cause of morphological damage of treated *C. albicans*. There was a statistical difference between untreated negative control and *Bacillus* sp. A2‐treated *C. albicans* (*p* < .001). ROS production and accumulation in *Bacillus* sp. A2‐treated *C. albicans* were lower compared to positive control of H_2_O_2_‐treated *C. albicans* but not statistically significant (*p* > .05) (Figure 6).

### 3.5 Mitochondrial membrane potential detection in treated *C. albicans*

**Figure 7:**
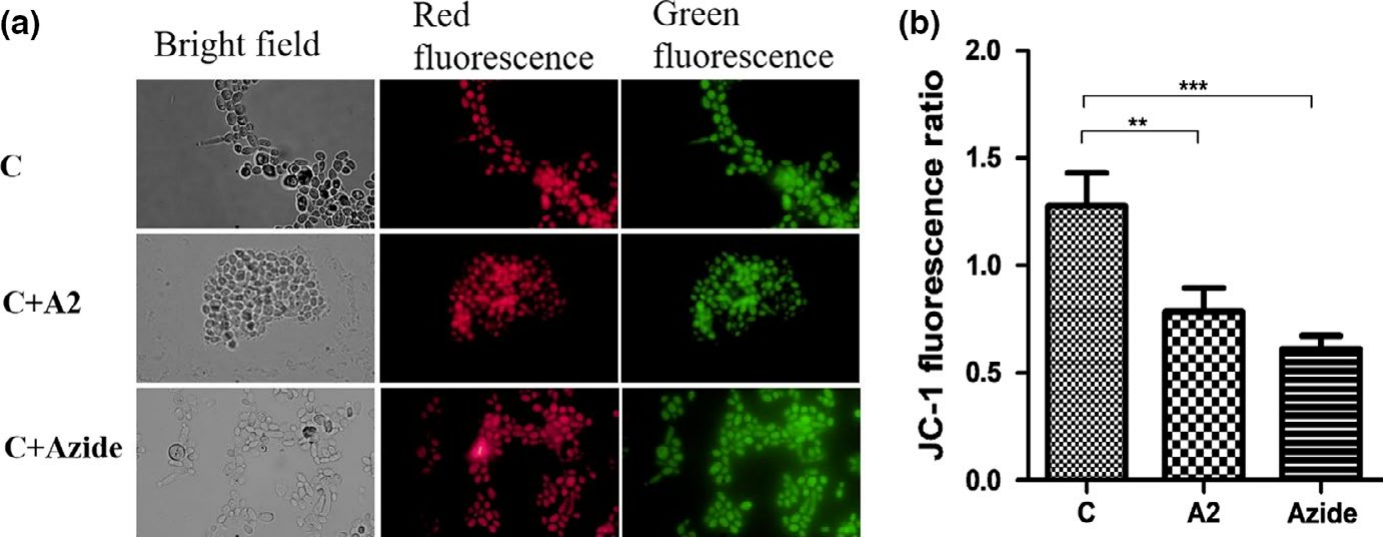
Measurement of mitochondrial membrane potential in treated *C. albicans* using JC‐1 fluorescent probe staining. (a) JC‐1 fluorescent probe‐stained *C. albicans*, (b) JC‐1 fluorescent *C. albicans* expressed as the ratio of aggregated JC‐1 (FL2 red fluorescence) to monomer (FL1 green fluorescence) intensity, (mean ± *SD*). C: negative control of untreated *C. albicans*, C + A2: *C. albicans* cocultured with *Bacillus* sp. A2 (24 hr), C + Azide: *C. albicans* treated with sodium azide (4 hr). The experiments were done three independent times, with three replicates each. A *p*‐value < .05 was considered to be significant, * denoted *p* < .05, ** denoted *p* < .01, and *** denoted *p* < .001

The integrity of mitochondria was assessed using mitochondrial membrane potential in which the ratio of aggregated JC‐1 (FL2 red fluorescence) to monomer of JC‐1 (FL1 green fluorescence) intensity was calculated (Figure 7a). Consequently, a decrease in the ratio meant mitochondrial depolarization. There was a statistical difference between the untreated negative control and *C. albicans* treated with or sodium azide, indicating *Bacillus* sp. A2 induced mitochondrial damage (*p* < .01) (Figure 7b).

## 4 DISCUSSION

Honey has the antimicrobial activity, which varies according to floral diversity (Koc et al., 2009) and influences phenols and flavonoid content (Moussa et al., 2012; Wahdan, 1998). Honeys’ high osmolarity is antibacterial. However, not antifungal since fungi possess high osmolarity glycerol (HOG) pathway (Hohmann, 2002; Wahdan, 1998). The presence of hydrogen peroxide in honey contributes to its antimicrobial activity (Matzen et al., 2018), but catalase honey still had antimicrobial activity (Feas, Iglesias, Rodrigues, & Estevinho, 2013), thus ruling out H_2_O_2_ as the sole inhibitor. Other inhibitors in honey are propolis, acids (Snyder et al., 2012), peptides, methylglyoxal, defensins, etc. In summary, the honey antimicrobial activity is not associated with a single factor. The role of antagonistic microorganisms and especially their mechanism of activity is understudied. Thus, in our study, we postulated that honey possessed antagonistic microorganisms against human pathogens. We validated this assumption by demonstrating that honey has microorganisms with antibacterial and antifungal activities, which has the potential of being harnessed as probiotics.

We isolated *Bacillus* sp. A2 of *B. amyloliquefaciens* group from honey with antimicrobial activity. *B. amyloliquefaciens* inhabit vast environments, which include; wastewater, air (Geeraerts, Ducatelle, Haesebrouck, & Van Immerseel, 2015); farm produce (A. Lee, Cheng, & Liu, 2017), honey (Zhao, de Jong, Zhou, & Kuipers, 2015), soil, rhizosphere (Lim et al., 2016), sea (Wang, Wu, Chen, Lin, & Yang, 2016), as an endophyte (White et al., 2014). *B. amyloliquefaciens* has been investigated for a variety of its beneficial properties, among them remediation of aquatic water; as biofertilizer (Chowdhury, Hartmann, Gao, & Borriss, 2015; Pretorius, van Rooyen, & Clarke, 2015; White et al., 2014), removal of mycotoxin from animal feeds (Chang, Wu, Wu, Dai, & Sun, 2015; A. Lee et al., 2017); as a probiotic for broilers (Ahmed et al., 2014; Y. Li et al., 2015); biocontrol agent against *B. dothidea* (X. Li et al., 2016), *Agrobacterium tumefaciens* (Ben Abdallah, Frikha‐Gargouri, & Tounsi, 2015), *Fusarium, Botrytis, Pythium*, and *Rhizoctonia* (Yuan et al., 2014); antimicrobial agent against *E. coli*, *S. aureus* (Ndlovu, Rautenbach, Vosloo, Khan, & Khan, 2017), *C. albicans* (Ndlovu, Rautenbach, Vosloo, Khan, & Khan, 2017; Wang et al., 2016), *Clostridium difficille* (Geeraerts et al., 2015), and *Listeria monocytogenes* (Lim et al., 2016); industrially for production of protease and amylase enzyme and as source of antimicrobials, for example, macrolactin A and E, bacillomycin D, (Yuan et al., 2014). The origin of *Bacillus* sp. isolated in honey might be intricate to establish considering its aforementioned habitats. Another complexity is that when bees collect water and nectar, they tend to come in contact with all these habitats. *B. amyloliquefaciens* is used commercially as biofertilizer and biocontrol agent in agriculture (Bai et al., 2014; Ben Abdallah et al., 2015). Interestingly, White et al. (2014) isolated *B. amyloliquefaciens* as a systemic endophyte in vanilla orchids *Vanilla phaeantha*. Therefore, the bacteria might have been picked by bees from any of these habitats, but it is fascinating if a plant biocontrol agent ends up on the plate! It is more intriguing if it culminates in being beneficial to both the plant and the secondary or tertiary consumer.

The isolated microorganism exhibited potent antimicrobial activity against *C. albicans* after 24 hr and *C. albicans*, *S. aureus*, and *E. coli* both drug‐resistant and drug‐sensitive after 48 hr of coculture. However, *P. aeruginosa* and *C. glabrata* did not show any sensitivity to the isolate in comparison with positive control *L. plantarum* and untreated negative control. We deduced that the bacterial isolate produced both antifungal and antibacterial compounds that were species‐specific, and the findings were similar to the previous reports (do Carmo et al., 2016). Our research work is interested in anti‐candida; therefore, we further probed the killing mechanisms, especially if it induced apoptosis in *C. albicans*. Apoptosis is outlined by a sequence of unique morphological changes which include; visible cell shrinkage, chromatin condensation, extensive plasma membrane blebbing, nuclear fragmentation, formation of apoptotic bodies. Apoptosis terminates with the decomposition of apoptotic bodies within the phagosome and complete recycling of the components (Eisenberg‐Lerner, Bialik, Simon, & Kimchi, 2009; Elmore, 2007). The results from *SEM* and TEM disclosed that *Bacillus* sp. A2 induced these morphological changes in *C. albicans*. However, apoptosis and necrosis have considerable overlap on the mechanism and morphologies; as a result, it was not possible to distinguish between the two mechanisms using microscopy. Consequently, determination of apoptosis was supported by additional apoptotic‐specific markers which include biochemical and cytological responses of an apoptotic cell precisely the accumulation of ROS and mitochondrial membrane potential (Elmore, 2007).

We first investigated whether the bacterial isolate could survive in low pH. The results confirmed that the lowest pH was 4.94 (Appendix S1), while the range of pH in honey is documented as 3.77–4.01. Thereafter, we suggested that *Bacillus* sp. A2 could survive in the honeys’ low pH. This low pH could not be attributed to the antimicrobial activity of *Bacillus* sp. A2 as a pH of 4.94 does not inhibit fungal pathogens because they are more resistant to lower pH up to 1.6–1.8 (Wahdan, 1998). Therefore, we had to investigate other factors that lead to the death of *C. albicans* when cocultured with *Bacillus* sp. A2.

We further discovered that *Bacillus* sp. A2 did not produce H_2_O_2_ exogenously (Appendix S1). It was essential to probe hydrogen peroxide production since it is converted to reactive oxygen species (ROS) such as hydroxyl free radicals and superoxide anion (J. D. Santos, Piva, Vilela, Jorge, & Junqueira, 2016; Verdenelli et al., 2014). Hydrogen peroxide‐producing microorganisms keep high oxido‐reduction potential in their habitat, which inhibits the multiplication of anaerobes such as (Verdenelli et al., 2014), *Giardia vaginalis*, *C. albicans*, and *Neisseria gonorrhea* (Kullisaar et al., 2002; Santos, Lima, Ruiz, Almeida, & Silveira, 2014). Hydrogen peroxide also induce cell stagnation and cell death (Hertzberger et al., 2014). However, *Bacillus* sp. A2 did not produce hydrogen peroxide, which meant that it had other mechanisms of causing cell death.

Probiotics, for example, *Lactobacillus bulgaricus* and *Bifidobacterium longum*, protect from infection by producing H_2_O_2_ (Pridmore, Pittet, Praplan, & Cavadini, 2008). Of interest, *Bacillus* sp. A2 did not produce H_2_O_2_ exogenously, but it induced production and accumulation of ROS inside *C. albicans*. Furthermore, it was observed that *Bacillus* sp. A2 decreased mitochondrial membrane potential. Both ROS accumulation and decreased membrane potential are well‐known biochemical and cytological responses of programmed cell death (PCD) such as apoptosis (Elmore, 2007), or at very high concentrations induce necrosis (Avery, 2011). In addition, accumulated ROS inflict oxidative damage upon essential biomolecules such as nucleic acids (Yakes & Van Houten, 1997), proteins (Cabiscol, Piulats, Echave, Herrero, & Ros, 2000), and lipids (Biliński, Litwińska, Błaszczyński, & Bajus, 1989). Reactive oxygen species comprise of (ROS) superoxide radical $\left(O_{2}^{.‐}\right)$, H_2_O_2_, and hydroxyl radical (OH^·^). These ROS play a role in the production of reactive nitrogen species (RNS), which are nitric oxide radical (NO^·^) and peroxynitrite (ONOO^−^), and thus aggravate the cell condition. Consequently, ROS is sufficient to induce PCD via apoptosis, necrosis, or autophagy.

In conclusion, our study demonstrated that honey has antagonistic microorganisms with antimicrobial property. We validated this assumption by demonstrating that one isolate out of fourteen screened from honey had antimicrobial activities. The isolate was identified as *Bacillus* sp. A2 and had antimicrobial activity against *E. coli*, *S. aureus*, *C. glabrata*, and *C. albicans*. Isolate *Bacillus* sp. A2 induced apoptosis in *C. albicans* by promoting the production and accumulation of ROS in *C. albicans* and damaging mitochondria, which is a vital organelle involved in energy production. Therefore, we conclude that honey is a candidate food that has the potential as a source of probiotic. Accordingly, we recommend honey microorganisms to be explored as a high potential antimicrobial source and as a probiotic. Second, we recommend an investigation on the source of these beneficial microorganisms in honey. Third, an investigation if the presence of antagonistic microorganisms is universal in honey. Finally, the results implicated benefit microorganism or probiotics contribute the honey's regulation to the microbiota.

## CONFLICT OF INTEREST

There is no conflict of interest to declare.

## ETHICAL APPROVAL

This article does not contain any studies with human participants or animals performed by any of the authors.

## Supporting information

Supplementary Material

## Data Availability

The nucleotide sequence for the identification of the microorganisms isolated 16S rRNA for this study is deposited in NCBI, GenBank submission: MK540476 (link https://www.ncbi.nlm.nih.gov/search/all/?term=MK540476).
