# Supplementary material for: Antimicrobial and mechanism of antagonistic activity of Bacillus sp. A2 against pathogenic fungus and bacteria: The implication on honey's regulatory mechanism on host's microbiota
Source: Food Sci Nutr. 2020 Jul 20;8(9):4857–67. doi: 10.1002/fsn3.1770 (PMC7500754; doi:10.1002/fsn3.1770)
Supplement: Supplementary file 1 — Supplementary Material [file FSN3-8-4857-s001.docx]

**SUPPLEMENTARY MATERIALS**

**1.0 Supplementary methods**

**1.1 Antimicrobial activity confirmation by liquid co-culture**

Liquid co-culture (Kosgey et al., 2019; Matsubara, Wang, Bandara, Mayer, & Samaranayake, 2016) were done in order to confirm the antimicrobial activity of honey isolate. The honey isolate (10^8^) were co-cultured with *C. albicans* (10^5^ CFU/mL) in NA: YPD (1:1) broth at 37°C for 24 hours in an aerobic incubator (180rpm). One milliliter of the cultures was retrieved was sampled at 1, 2, 4, 8, 12, 24, 48, and 72 hours and change in pH was obtained by measuring with pH meter.

**1.2 Detection of hydrogen peroxide (H_2_O_2_) producers**

Qualitative plate assay was used in which chromogen (TMB: 3,3',5,5'-tetramethyl-benzidine) (0.25g/l) and peroxidase enzyme (EC 1.11.1.7/horseradish peroxidase type II) (0.01g/l) was added to nutrient agar (NA). Corn starch 2%, magnesium sulfate anhydrous 0.06%, and manganese sulfate monohydrate 0.012% were added to NA to enhance blue color formation. A2 isolate negative control L and positive control *L. acidophilus* (Santos et al., 2016) were adjusted to 10^6^ cells/ml and inoculated on TMB agar. The plates were incubated at 37°C for 24 hours in an anaerobic jar. The plates were then exposed to air for about 30 minutes; until positive control changed color. Colonies with blue color were considered H_2_O_2_ producers.

**2.0 Supplementary results**

**2.1 Change in pH with time**

Change in pH of media inhibits pathogens. Thus, we evaluated if this was the mechanism of antimicrobial activity of *Bacillus* sp. A2. The results showed a shift in pH from 6.5 (1 hour) to 4.9 being the lowest recorded at 48 hours. We concluded that pH might be one of the mechanisms of antimicrobial activity of *Bacillus* sp. A2 but not the sole reason given that fungal pathogens can withstand this pH (Fig S1a). Therefore, we sought to establish other mechanisms of antimicrobial activity of *Bacillus* sp. A2.

**2.2 Detection of extracellular production of hydrogen peroxide**

We sought to determine if they produce H_2_O_2_ exogenously, and found out that the bacteria were non-producers, while the positive control *L. acidophilus* was positive for H_2_O_2_ as it created a blue pigmentation in TMB media. Thus, we deduced that the bacteria could have alternative mechanisms of activity other than exogenous production of hydrogen peroxide (Fig.S1b).


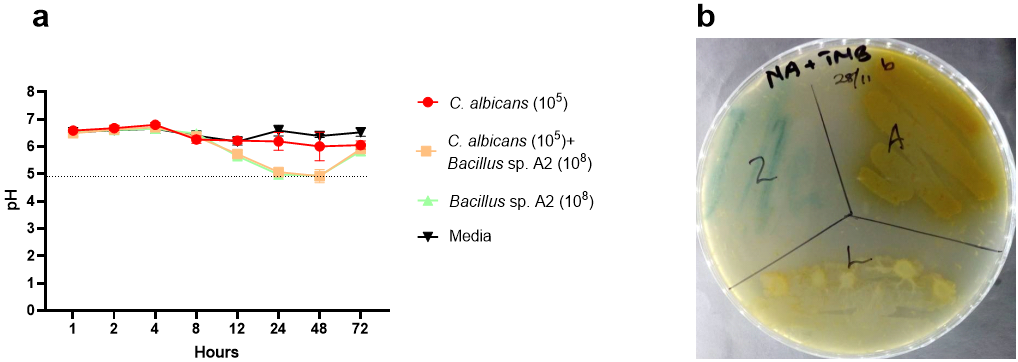


**Fig. S1** Demonstrates the change in pH over time, and exogenous production of H_2_O_2_ on TMB media. [a] Shows the variation in pH over time for different treatments. *C. albicans* (10^5^)- untreated *C. albicans*, *C. albicans* (10^5^) + *Bacillus* sp. (10^8^)- *C. albicans* treated with *Bacillus* sp. A2, *Bacillus* sp. (10^8^)- pure culture of *Bacillus* sp. A2 (10^8^) and lastly media; 1:1 mixture of YPD and nutrient broth (mean ± sd). The dotted line indicated the lowest measured pH. [b] H_2_O_2_ production by *Bacillus* sp. A2 and positive control *L. acidophilu*s (2) & negative control L

**References**

Kosgey, J. C., Jia, L., Fang, Y., Yang, J., Gao, L., Wang, J., . . . Wekesa, V. J. J. o. m. m. (2019). Probiotics as antifungal agents: Experimental confirmation and future prospects.

Matsubara, V. H., Wang, Y., Bandara, H. M. H. N., Mayer, M. P. A., & Samaranayake, L. P. (2016). Probiotic lactobacilli inhibit early stages of Candida albicans biofilm development by reducing their growth, cell adhesion, and filamentation. *Applied Microbiology and Biotechnology, 100*(14), 6415-6426. doi:10.1007/s00253-016-7527-3

Santos, C. M. A., Pires, M. C. V., Leão, T. L., Hernández, Z. P., Rodriguez, M. L., Martins, A. K. S., . . . Nicoli, J. R. (2016). Selection of Lactobacillus strains as potential probiotics for vaginitis treatment. *Microbiology, 162*(7), 1195-1207. doi:doi:10.1099/mic.0.000302
